# A Case of Cerebral Cortical Encephalitis

**DOI:** 10.1002/acn3.70368

**Published:** 2026-03-15

**Authors:** Sixiao Liu, Kunqian Ji, Wei Wu, Wei Li

**Affiliations:** ^1^ Department of Neurology, Shandong Key Laboratory of Mitochondrial Medicine and Rare Diseases Research Institute of Neuromuscular and Neurodegenerative Diseases, Qilu Hospital, Cheeloo College of Medicine, Shandong University Jinan Shandong China

**Keywords:** anti‐NMDAR encephalitis, cortical encephalitis, leptomeningeal hyperintensity, orolingual dyskinesia

## 1 Summary of Case

A 60‐year‐old previously healthy woman presented with a 2‐month history of progressive cognitive decline and behavioral abnormalities, including mutism and purposeless movements. Neurologic examination revealed impaired consciousness, negativistic behavior, and stereotyped orolingual dyskinesias. Brain MRI showed bilateral frontal–parietal and left temporal leptomeningeal hyperintensities on T2‐FLAIR and DWI. CSF analysis revealed 28 WBCs/μL (95% lymphocytes), normal protein and glucose, and strongly positive anti‐NMDAR antibodies (CSF titer 1:100; serum 1:10). EEG showed moderate diffuse slowing. Infectious panels, oligoclonal bands, and other autoimmune antibodies were negative. Tumor screening showed no malignancy but identified a right adnexal unilocular cyst. Based on imaging, clinical, and immunologic findings, anti‐NMDAR encephalitis was diagnosed. The patient was treated with high‐dose intravenous methylprednisolone and IVIG, resulting in marked clinical improvement. Follow‐up MRI at 3 months showed near‐complete resolution of abnormalities, and she achieved full functional recovery by 6 months.

## 2 Diagnosis

Cerebral cortical encephalitis in anti‐NMDAR autoimmune encephalitis.

## 3 Take‐Home Points

**FIGURE 1:**
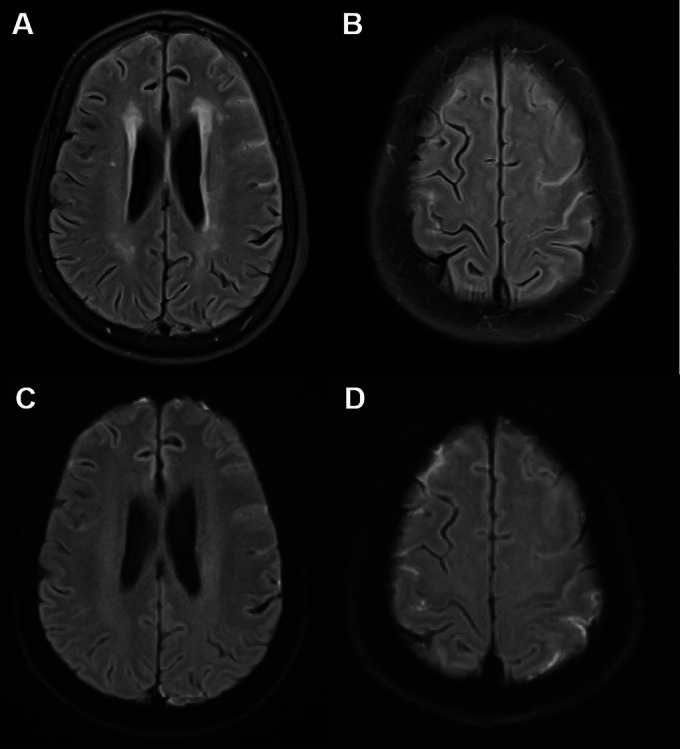
Axial FLAIR MRI sequences (A and B) and DWI (C and D) showing hyperintense lesions in the bilateral frontal–parietal and left temporal leptomeninges.

**FIGURE 2:**
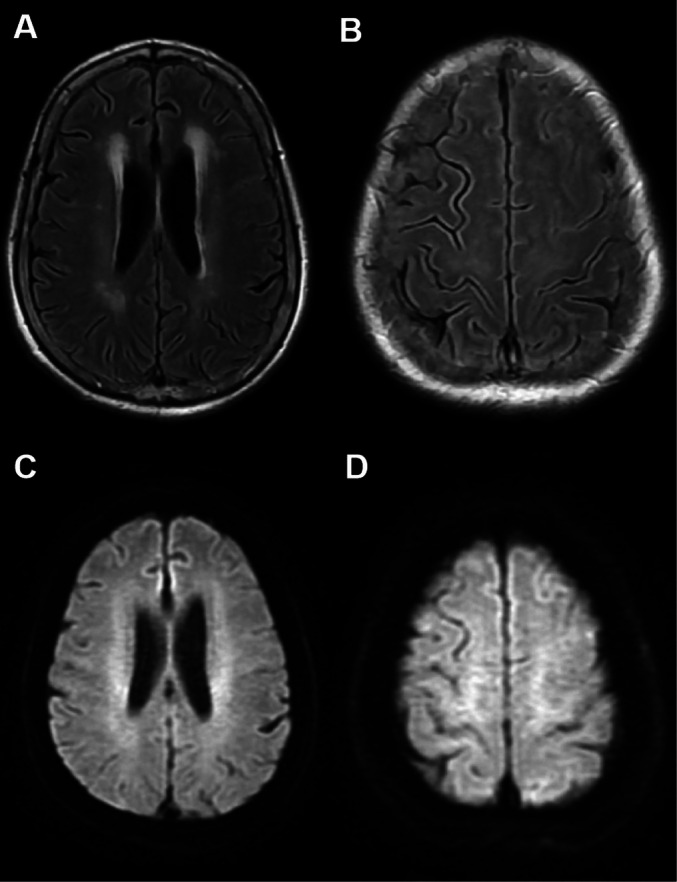
Axial FLAIR MRI sequences (A and B) and DWI (C and D) showing near‐complete resolution of leptomeningeal hyperintensities.

- Anti‐NMDAR encephalitis is an autoimmune disorder characterized by neuropsychiatric symptoms, seizures, and movement abnormalities. While MRI is normal in approximately 50% of cases, some exhibit T2‐FLAIR hyperintensities in the cortex or leptomeninges [1, 2].
- This case showed bilateral, multilobar leptomeningeal hyperintensity (Figure 1) resembling the FLAMES pattern; differentiation from MOG‐associated encephalitis is essential, as FLAMES typically features unilateral cortical involvement and positive MOG antibodies [3, 4].
- Definitive diagnosis requires detecting anti‐NMDAR antibodies in the CSF, which is more sensitive and specific than serum testing (~99% vs. ~68%) [2]. CSF findings often show lymphocytic pleocytosis with normal glucose/protein and negative oligoclonal bands.
- EEG may show moderate diffuse slowing, reflecting global cortical dysfunction and supporting diagnosis [1].
- Anti‐NMDAR encephalitis is often paraneoplastic, most commonly associated with ovarian teratomas. While no tumor was confirmed in this case, a right adnexal unilocular cyst was noted [2].
- Early immunotherapy with high‐dose corticosteroids and IVIG can lead to rapid clinical and radiological improvement. MRI at 3 months showed near‐complete lesion resolution (Figure 2) [5].
- Autoimmune encephalitis may mimic viral or demyelinating diseases; atypical imaging should prompt thorough clinical and antibody evaluation.

## 4 To Be Completed by the Editorial Office

See the discussion, including references, in the full case published here: https://interactn.org/2026/03/03/case‐57‐cerebral‐cortical‐encephalitis/.

## Author Contributions

**Sixiao Liu:** writing – original draft and data analysis. **Kunqian Ji:** writing – review and editing. **Wei Wu:** supervision. **Wei Li:** supervision.

## Funding

The authors have nothing to report.

## Conflicts of Interest

The authors declare no conflicts of interest.

## Data Availability

The data that support the findings of this study are available on request from the corresponding author. The data are not publicly available due to privacy or ethical restrictions.
